# Navigating the Storm: Understanding Affective Instability and Its Implications

**DOI:** 10.1192/j.eurpsy.2025.1929

**Published:** 2025-08-26

**Authors:** A. F. Reis

**Affiliations:** 1 Psychiatry and Mental Health, Unidade Local de Saúde da Arrábida, Setúbal, Portugal

## Abstract

**Introduction:**

*Affective instability* (AI) is a psychophysiological symptom reported in many neurological and psychiatric conditions. It has assumed more relevance in the psychiatric literature as a criterion for borderline personality disorder (BPD). Although extensively clinically used, its definition remains vaguely defined, and it ends up being used interchangeably with *affective lability* or *emotional dysregulation*, and it is often mistaken for *mood lability*, as described in bipolar disorders. To accurately diagnose this symptom and document variations in emotional experiences, it is essential to identify the factors associated with AI.

**Objectives:**

We aim to review the current definitions and conceptualizations of AI to provide more accurate use of the term.

**Methods:**

Narrative literature review.

**Results:**

Current definitions of AI highlight the oscillation of emotions, often described as a series of intense emotional highs and lows that can shift within hours or even minutes, making it challenging for individuals to maintain a stable emotional baseline, significantly affecting an individual’s relationships, self-identity, and coping mechanisms. It is a complex construct, encompassing affective valence, affect amplitude, affective shifting with random patterning, reactivity thresholds to environmental triggers, and affective dyscontrolled modulation. Neurobiological research suggests that dysregulation in emotional processing areas of the brain, such as the amygdala and prefrontal cortex, may contribute to these rapid emotional shifts.

**Conclusions:**

AI is a multifaceted construct with significant implications for mental health. The current definitions and conceptualizations underscore the complexity of emotional regulation and the need for a holistic approach to understanding and treating individuals experiencing these emotional fluctuations. Continued research into the neurobiological, psychological, and environmental underpinnings of affective instability will enhance the understanding of this phenomenon and improve treatment strategies for affected individuals.

**Disclosure of Interest:**

None Declared

